# Space-time acoustic coding metasurfaces with scattering reduction characteristics

**DOI:** 10.1016/j.isci.2025.112402

**Published:** 2025-04-09

**Authors:** Sheng He, Wenkang Cao, Kaiping Nie, Jinsong Ye, Jie Hu, Liting Wu

**Affiliations:** 1 School of Mechanical Engineering, Guizhou University, Guiyang 550025, China; 2 School of Information and Communication Engineering, Nanjing Institute of Technology, Nanjing 211167, China

**Keywords:** physics, Acoustics

## Abstract

In recent years, constructing space-time joint modulation acoustic metasurfaces with harmonic manipulation capabilities to achieve excellent scattering reduction within three-dimensional (3D) space has remained a significant challenge. Here, space-time acoustic coding metasurfaces (STAMs) with harmonic manipulation in 3D space are demonstrated, which consist of two types of sub-wavelength unit cells. By changing acoustic impedance of proposed unit cells, the phase difference between these unit cells can maintain 180° in the operation frequency. Both theoretical calculations and numerical simulations verified that compared with acoustic coding metasurfaces of the same size with spatial coding sequence M1(0000 …), the maximum sound pressure level (SPL) of the STAM can decrease about 22 dB. The proposed space-time joint modulation mechanism can provide a method for achieving flexible acoustic wavefront manipulation, which has potential applications in acoustic stealth technology, noise control, and other relevant applications.

## Highlights

• A time-modulation strategy for flexible regulation of harmonic energy is proposed
• STAM unit cell with reflection phase varying with acoustic impedance
• STAM exhibits excellent scattering reduction capability for acoustic wave energy

## Graphical abstract

**Figure:**
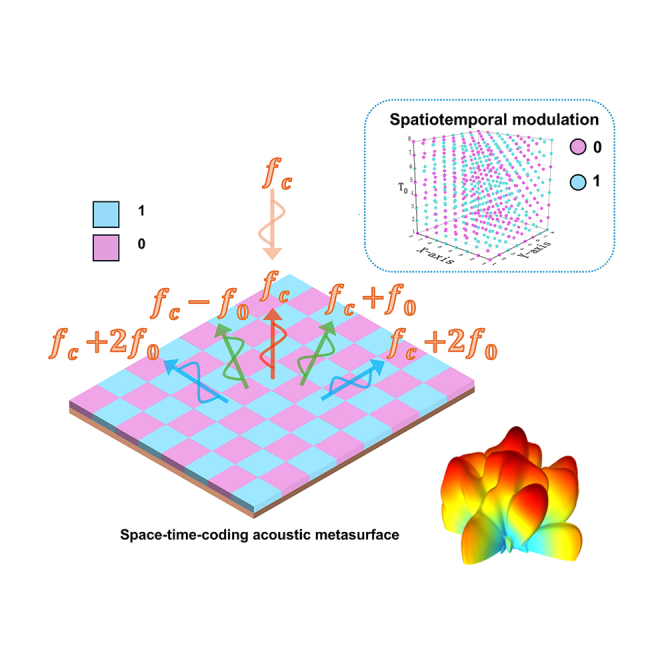

## Introduction

In recent decades, acoustic metamaterials, which are man-made structures with sub-wavelength thickness and control acoustic waves in a distinctive manner, have witnessed significant advancements across a range of disciplines.^1^^,^^2^^,^^3^ Subsequently, two-dimensional (2D) acoustic metasurfaces, arranged by sub-wavelength unit cells, are selected by researchers due to their excellent characteristics, including compact structure, low loss, and flexible wavefront manipulation.^4^^,^^5^^,^^6^ Therefore, a wide range of intriguing functions and phenomena in these fields such as diffuse reflection,^7^^,^^8^ beam focusing,^9^ asymmetric transmission,^10^ near-perfect absorption,^11^^,^^12^^,^^13^ acoustic stealth,^14^^,^^15^ and acoustic holography^16^ are implemented. Specially, the arbitrary manipulation of scattering acoustic waves has always been a hot topic of acoustic metasurfaces, which are mainly based on acoustic diffuser^17^ or absorber^18^ to reduce the echo power intensity. In fact, acoustic diffuser metasurface can disperse incoming energy in various directions.^7^ Therefore, the diffuse reflection phenomenon can obviously reduce the backscattering intensity of an object. However, the majority of the existing acoustic metasurfaces with scattering reduction characteristics focus on adjusting spatial phase distributions of metasurfaces, whereas the time dimension has not been exploited adequately.

Furthermore, coding metamaterials and metasurfaces, which have made rapid progress since they were proposed in 2014, can manipulate electromagnetic (EM) waves to realize different functions by controlling coding sequences of “0” and “1” bits that correspond to the 0° and 180° phase response.^19^ A lot of coding metasurfaces with advanced functionalities have been proposed, such as multi-beam modulation,^20^ orbital angular momentum beam,^21^ and full-space wavefront manipulations.^22^^,^^23^ In order to achieve more sophisticated operation, time modulation of reflection or transmission coefficient is exploited in combination with space coding metasurfaces^24^^,^^25^^,^^26^ for more flexible beam steering,^27^^,^^28^ scattering control,^29^^,^^30^ and multi-frequency independent manipulation.^31^^,^^32^^,^^33^ Subsequently, as the counterpart for EM coding metasurfaces, acoustic coding metasurfaces were proposed in 2017 using coding sequences of designed Boolean elements, and it demonstrates that coding metasurfaces can flexibly manipulate acoustic waves.^34^ Thereafter, another binary acoustic coding metasurface with perfect anomalous reflective and refractive properties has been proposed.^35^ In the same year, Xie et al. proposed multi-bit coding acoustic metasurfaces to manipulate the reflection of acoustic waves, demonstrating wave branching and acoustic directional propagation.^36^ In addition, another reflection-type acoustic coding metasurface is proposed to obtain broadband acoustic focusing lenses.^37^^,^^38^ Recently, an arbitrarily curved 1-bit coding metasurface has been proposed to steer the far-field acoustic waves.^39^ Although the aforementioned acoustic coding metasurfaces have achieved rich functions, unfortunately, these acoustic coding metasurfaces have been designed with fixed functions. Specially, only space coding has been considered in these acoustic coding metasurfaces, and the time dimension has not yet been utilized.

Recently, space-time modulation acoustic metamaterials and metasurfaces have become an active research area, enabling a range of functionalities such as realize nonreciprocal transport,^40^ orbital angular momentum multiplexing,^41^^,^^42^ and rotational Doppler effect.^43^ Among that attractive research of utilizing time dimension to control acoustic waves, recently, spatiotemporally modulated acoustic metasurfaces with sound diffusion function have been proposed to improve diffusivity of the backscattered field by introducing spatiotemporal modulation of the surface acoustic admittance.^44^ Furthermore, the concept of space-time coding metasurface is introduced into acoustic field by Fakheri et al. in 2021. By transferring the energy of the carrier acoustic signal to a series of harmonic components, several different scattering functions and beam steering capabilities have been realized by controlling the temporal and space coding sequences.^45^ In the meantime, nearly, the spatiotemporally modulated acoustic metamaterials that support nonreciprocal sound steering are implemented by Chen, which can realize programmable dynamic manipulation of the acoustic impedance.^46^ Although the aforementioned research results have achieved good results in harmonic energy manipulation, nearly all of the acoustic metasurfaces with time-modulation function can achieve wavefront manipulation within 2D space. Especially, in order to further improve the performance of scattering reduction and flexibly control acoustic waves, the space-time joint modulation should be exploited within three-dimensional (3D) space by utilizing both the space and time dimensions, which is also urgently required in practical applications, such as target stealth and camouflage.

In this paper, the space-time acoustic coding metasurfaces (STAMs) with harmonic manipulation in 3D space are proposed, which consist of two kinds of sub-wavelength unit cells with phase difference of 180° achieved by changing acoustic impedance of the proposed ones. Subsequently, several illustrative examples are presented to demonstrate the ability of the proposed STAM to transfer the energy of incident acoustic waves to other harmonics and randomly disperse it in the upper half space via adjusting space-time coding sequences of the STAM. The proposed space-time joint modulation mechanism extends the application range of acoustic metasurfaces with potential values in acoustic cloaking,^47^ noise control,^48^ and beamforming scenarios.^45^

## Results

### Design principle of the STAMs

**Figure 1:**
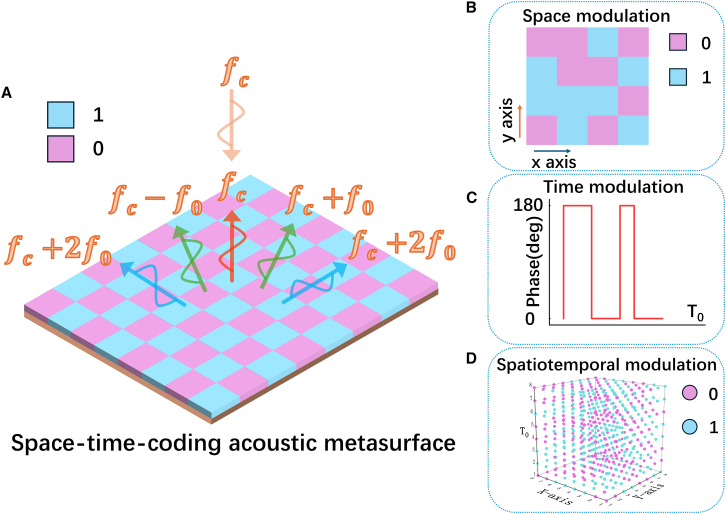
Schematic of the STAM (A) Conceptual illustration of the proposed STAM, whose scattering pattern can be modulated according to space-time coding sequences. $f_{c}$ is the carrier frequency, and $f_{0}$ is the modulation frequency. (B and C) The coding sequences of space modulation and time modulation. (D) The coding sequence of the spatiotemporal modulation by utilizing both space and time dimensions.

In order to improve the performance of acoustic scattering reduction based on space-time joint modulation mechanism, the STAMs which consist of an array of M × N adjustable coding elements are demonstrated in Figure 1A. The STAM is divided into two layers, of which the upper layer consists of the pre-designed unit cells with reflection phase varying with time coding sequences and the lower layer is a perfect rigid boundary that reflects the incident acoustic waves completely. Here, the reflection phase of the unit cells can be periodically switched according to the “0/1” space/time coding sequences in Figures 1B and 1C. Here, the 0 or 1 bit represents the reflection phase 0° or 180°, respectively. Hence, for an arbitrary space-time acoustic coding sequence in Figure 1D, the scattering pattern of the proposed STAM at any harmonic frequency can be obtained. Furthermore, the reflection phase of all coding elements is periodically switched, according to the space-time modulation coding sequences demonstrated in Figure 1D.

### Theoretical analysis

**Figure 2:**
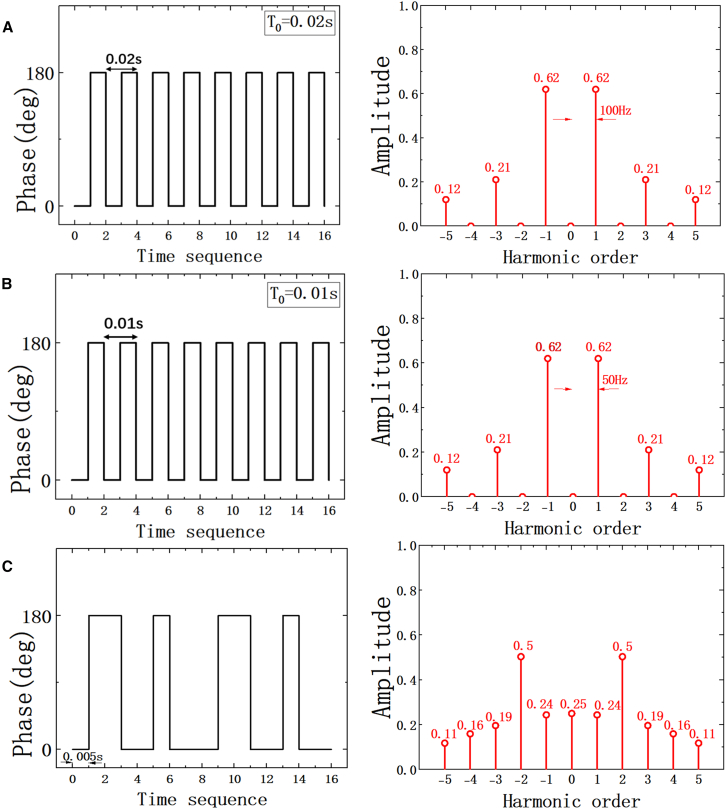
The theoretical analysis of harmonic amplitude distributions of the proposed STAM (A) Time coding sequence 0101 …, L = 2 and T0 = 0.01 s. (B) Time coding sequence 0101 …, L = 2 and T0 = 0.02 s. (C) Time coding sequence 01100100 … and L = 8.

According to the theoretical analysis in method details, far-field scattering patterns of the STAM at any harmonic frequencies can be achieved by reasonably and accurately controlling the space-time coding sequences. To illustrate this ability, illustrative examples are shown in Figure 2, according to Equation 5. Here, reflection phases of coding elements are switched according to time coding sequences in Figures 2A–2C (left insets), while the space modulation of reflection phases is invariable. As can be observed, Figures 2A and 2B (right insets) further show the harmonic amplitude distribution of the STAM with time modulation $T_{0}$ = 0.02 and 0.01 s, respectively, which are equivalent to modulation frequency $f_{0}=50$ and 100 Hz. Specifically, when the reflection phase varies periodically either 0° or 180° by the time coding sequence 0101 …, the $0^{th}$-order harmonic energy is almost eliminated, which is attributed to the cancellation of the signals of the inverted reflectivity in each period. Moreover, the harmonic amplitude of ${\pm 1}^{\text{th}}$ harmonics reach 0.62, the harmonic amplitude of ${\pm 3}^{\text{th}}$ harmonic reaches 0.21, and the harmonic amplitude of ${\pm 5}^{\text{th}}$ harmonic reaches 0.12, which indicates that the most energy of incident waves is shifted to ${\pm 1}^{\text{th}}$ harmonics. The aforementioned results demonstrate that the energy of incident waves can be manipulated and transferred to odd order harmonics via time coding sequences 0101 …. In order to further obtain more randomly distributed reflection intensity spectrum, the time coding sequence 01100100 is provided in Figure 2C (left inset). Here, the minimum time-modulation interval T_0_ is 0.005 s. As is shown in Figure 2C (right inset), the energy of incident waves is shifted to different order harmonics by utilizing an irregular time coding sequence, and the reflection intensity spectrum exhibits approximately random feature. Compared with the aforementioned intensity spectrums with regular time coding sequences, the maximum amplitude of harmonics is reduced to 0.5, which can be observed in ${\pm 2}^{\text{th}}$-order harmonics. In addition, the harmonic amplitudes of the ${\pm 3}^{\text{th}}$, ${\pm 4}^{\text{th}}$, and ${\pm 5}^{\text{th}}$ orders are 0.19, 0.16, and 0.11, respectively. Furthermore, by increasing the length of time coding sequences and optimizing ones, the intensity spectrum distribution can become more random and even, as shown in Figure S1.

### Numerical simulation of the unit cell

**Figure 3:**
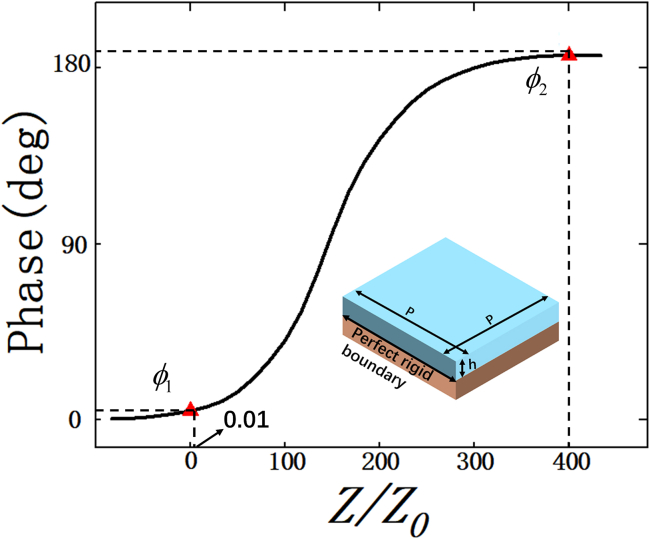
Simulated reflection phase variation of unit cells as a function of relative acoustic impedance *Z/Z0* Inset: unit cell of the STAM, which consists of two layers. The reflection phase can cover 180° with the relative acoustic impedance of upper layer varying from 0.01 to 400 and a perfect rigid boundary at bottom to obtain total reflection.

In order to verify the design principles of the proposed STAM in Figure 1 and theoretical results of harmonics amplitude distributions in Figure 2, a unit cell is proposed, as shown in Figure 3 (inset), which consists of two layers. Here, the thickness h of the proposed unit cells is 3 mm, and the periods of ones are 25 mm along both *x* and *y* directions. The acoustic impedance *Z* of the upper layer (Z=ρc; *ρ* and c is mass density and speed of sound of it, respectively.) can be changed to realize the required reflection phase 0° and 180°. It can be pointed out that the piezoelectric membrane can be used as the unit cell. The density of the membrane can be controlled in real time by varying the voltage of these membranes, which translates into controlling the acoustic impedance of unit cells.^46^^,^^49^ Furthermore, the time-varying phase also can be realized by dynamically adjusting geometrical parameters of unit cells. As reported by ourself before,^50^ the unit cell consists of two cylindrical cavities with distinct depths and diameters, and the end of unit cells is connected to an array of motors. The desired time-varying reflection phase can be continuously modulated via a motorized control system. Here, in order to validate the reflection characteristics of the proposed unit cell, Finite-Element Analysis software COMSOL Multiphysics 6.2 with the pressure acoustic module is utilized to calculate the reflection phase and acoustic pressure distributions of the proposed STAM with different space-time coding sequences. The background medium is air with mass density $\rho_{0}=1.29kg/m^{3}$ and speed of sound $c_{0}=343m/s$. In addition, periodic boundary conditions are around the unit cell. By simulating the near-field and far-field acoustic pressure field distribution, a plane wave is incident along -*z* direction with 1 Pa amplitude. The unit cell and STAM are surrounded by a perfectly matched layer to reduce unnecessary reflections.

As shown in Figure 3, by adjusting the relative acoustic impedance *Z/Z0* ($Z_{0}=\rho_{0}c_{0}$) of the unit cell, the reflection phase gradually increases from 0° to 180°. To be specific, the phase difference is exactly 180° by selecting the phase $\emptyset_{1\;}$ (relative impedance of 0.01) and phase $\emptyset_{2\;}$ (relative impedance of 400). In addition, the bottom layer is the perfect rigid boundary to ensure total reflection of acoustic waves.

**Figure 4:**
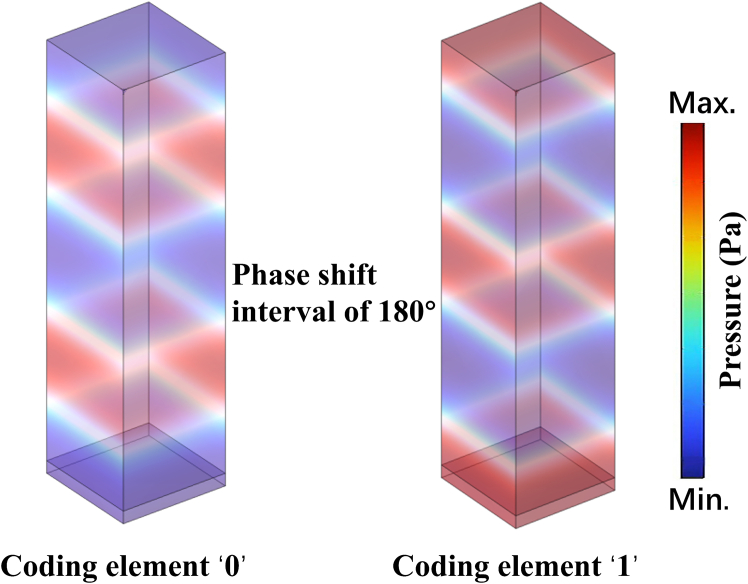
Scattered pressure field distribution for coding element “0” and coding element “1” at 6,860 Hz under a normal incident plane wave

To demonstrate the reflective behaviors of these unit cells, the simulated results of scattered pressure fields under the incidence of a plane wave at 6,860 Hz are shown in Figure 4. The 180° phase shift interval between coding element “0” and coding element “1” is obtained with the relative acoustic impedance of 0.01 and 400, respectively. Due to the proposed coding elements with an antiphase characteristic of reflected waves, when these elements are distributed with an accurately arranged space coding sequence, the strong interference phenomenon between ones can achieve obvious scattering reduction.^7^^,^^51^ Hence, the STAM with space-time joint modulation mechanism can obtain better scattering reduction performance, which is certified in the following part.

**Figure 5:**
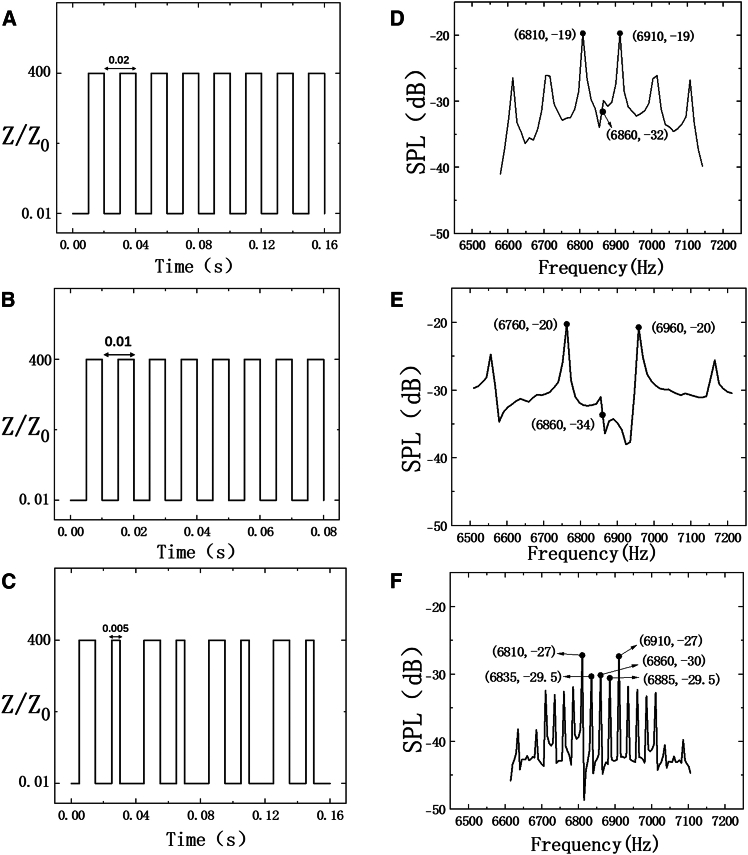
The time-varying relative acoustic impedance *Z/Z0* (A and B) Time period (A) 0.02 s and (B) 0.01 s. (C) The randomly varying relative acoustic impedance *Z/Z0* with minimum time length 0.005 s, which is consistent with the time coding sequence in Figure 2C. (D–F) The corresponding normalized SPL harmonic intensity distributions of the proposed STAM.

In order to prove the theoretical analysis results in Figure 2, the relative acoustic impedance *Z/Z0* is time varying, according to the different time coding sequences in Figures 5A–5C, which is consistent with Figures 2A–2C (left insets), while the space modulation of relative acoustic impedance is also invariable. As can be observed in Figure 5D, when the modulation frequency is 50 Hz, the energy of the incident acoustic wave is shifted to other frequencies with the most energy dispersed to 6,810 and 6,910 Hz. To be specific, the normalized sound pressure level (SPL) is −32 dB at the fundamental frequency 6,860 Hz and is −19 dB at both ${\pm 1}^{th}$ harmonics (6,810 and 6,910 Hz). Similarly, when the modulation frequency is 100 Hz, the energy of incident acoustic waves is shifted to 6,760 and 6,960 Hz. Specifically, the normalized SPL is −34 dB at 6,860 Hz and −20 dB at both 6,760 and 6,960 Hz, as shown in Figure 5E. Both of the aforementioned examples successfully demonstrate that the energy of incident acoustic waves can be moved to the corresponding odd order harmonics, which is in strong agreement with the analytical results in Figures 2A and 2Bb. Moreover, when relative acoustic impedance *Z/Z0* is randomly varying (Figure 5C), which is consistent with the time coding sequence in Figure 2C, the most energy of incident acoustic waves is shifted to other frequencies, as shown in Figure 5F. In detail, the normalized SPL is −30 dB at 6,860 Hz, while it is −29.5 dB at both 6,835 and 6,885 Hz as well as −27 dB at 6,810 and 6,910 Hz. Hence, a similar white noise spectrum can be achieved via introducing randomly varying acoustic impedance to the STAM.

### Numerical simulation of STAM

**Figure 6:**
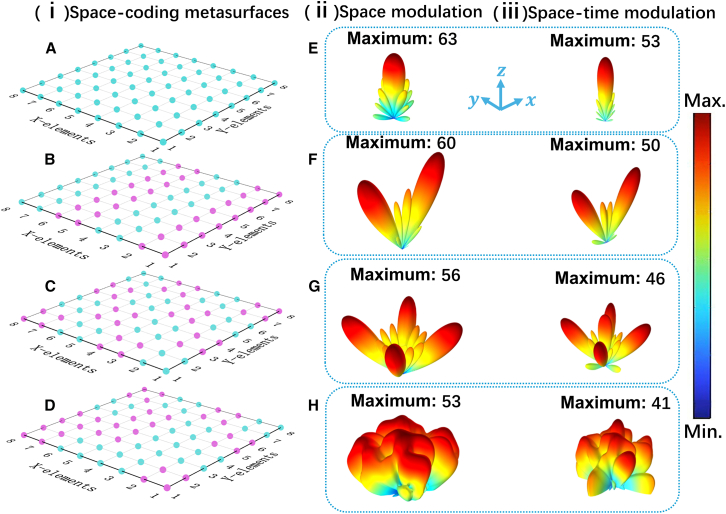
3D far-field SPL scattering patterns of STAM and ACM with different space coding sequences (A) The space coding sequence M1. (B) The space coding sequence M2. (C) The space coding sequence M3. (D) The space coding sequence M4. (E–H) ii: The 3D far-field SPL scattering patterns of ACM with corresponding space coding sequences and time-invariant acoustic impedance at 6,860 Hz. iii: The 3D far-field SPL scattering patterns of the proposed STAM at 6,860 Hz by combining corresponding space coding sequences with the same time coding sequence in Figure 5C.

Now, to prove the aforementioned mechanism combining space and time modulation at the same time, several STAMs consist of 4 × 4 coding elements sharing the same time-varying acoustic impedance in Figure 5C, while different space coding sequences in Figures 6A–6D are constructed. Here, each coding element contains 2 × 2 unit cells. As a comparison, the far-field SPL scattering patterns of acoustic coding metasurfaces (ACMs) of the same size with STAM are shown in Figures 6E–6H (ii). Here, the ACMs have different space coding sequences in Figures 6A–6D and time-invariant acoustic impedance. In detail, Figure 6E shows the 3D far-field SPL scattering patterns of ACM and STAM with space coding sequence M1 (0000 …). Obviously, the single beams are achieved, and the scattering pattern of the ACM in Figure 6E (ii) is consistent with a rigid plate of the same size. In detail, it can be seen that the maximum SPL of ACM is 63 dB, while that of the corresponding STAM decreases to 10–53 dB. Next, as shown in Figure 6F, a double scattering beam pointing to a pre-designed direction in the upper half space can be generated when the ACM and STAM are encoded with the space coding sequence M2 (0101 …) in Figure 6B, and the reflection angles of ACM and STAM are both 29.9° with respect to the *z* axis that is highly consistent with the theoretical results 30° calculated by $\theta =\arcsin\left(\lambda /2L_{0}\right)$ with the period of coding elements $L_{0}$ = 100 mm. However, by comparing the 3D far-field SPL scattering patterns of ACM and STAM in Figure 6F, it is clear that the maximum SPL of STAM decreases 10 dB. Similarly, when the ACM and STAM are encoded with a checkerboard-like space coding sequence M3 (0101 … /1010 …) in Figure 6C, the four scattering beams in the direction of *φ* = 45°, 135°, 225°, and 315° are further produced in Figure 6G. It is clear that the maximum SPL of STAM decreases to 46 dB, comparing with that of ACM (56 dB). However, when the ACM and STAM are encoded with an approximately random space coding sequence M4 in Figure 6D, we can still observe that the maximum SPL of STAM further decreases to 41 dB, while that of ACM is 53 dB. The simulated 3D far-field SPL scattering patterns in Figure 6 prove that an obvious scattering reduction characteristic can be obtained. To be specific, compared with an ACM of the same size with spatial coding sequence M1(0000 …) in Figure 6E (ii), the maximum SPL of the STAM can decrease about 22 dB in the specular direction.

**Figure 7:**
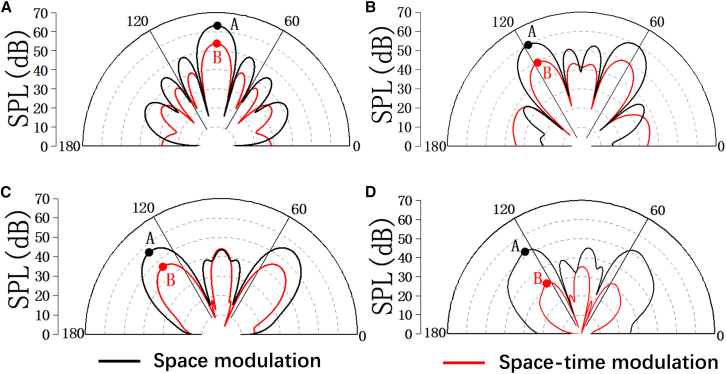
The corresponding 2D far-field SPL scattering patterns of the ACM and STAM with space coding sequences M1, M2, M3, and M4 in *xoz* plane (A) The corresponding 2D far-field SPL scattering patterns of the ACM and STAM with space coding sequences M1 in xoz plane. (B) The corresponding 2D far-field SPL scattering patterns of the ACM and STAM with space coding sequences M2 in xoz plane. (C) The corresponding 2D far-field SPL scattering patterns of the ACM and STAM with space coding sequences M3 in xoz plane. (D) The corresponding 2D far-field SPL scattering patterns of the ACM and STAM with space coding sequences M4 in xoz plane. Points A and B are the corresponding maximal SPL for ACM and STAM, respectively.

In addition, to further quantitatively investigate the performance of the acoustic backward scattering reduction, the corresponding 2D far-field SPL scattering patterns of ACM and STAM in *xoz* direction are shown in Figure 7. Here, points A and B are the corresponding maximal SPL for ACM and STAM, respectively. In comparison, the far-field SPL reduction between the ACM and STAM with space coding sequences M1, M2, and M3 in the direction of reflected waves is at least 10 dB, as illustrated in Figures 7A–7C. Furthermore, the energy of reflected waves of ACM and STAM is both redistributed to various directions by introducing the space coding sequence M4 in Figure 7D. Obviously, the 2D far-field SPL scattering patterns of the acoustic metasurface with space-time modulation are nearly inside the ACM with space modulation, which further confirms that the STAM can achieve an excellent acoustic backward scattering reduction. Finally, in order to further evaluate the intensity distribution of the reflected waves, the average SPL reduction between ACM and STAM in Figure 7D can be calculated by Equation 7. According to Equation 7, it can be found that the average SPL of STAM is approximately 22 dB lower than that of ACM. In addition, in order to demonstrate the operating bandwidth of the proposed STAM with space coding sequence M4, the corresponding 3D far-field SPL scattering patterns at 6,810, 6,835, 6,885, and 6,910 Hz are shown in Figure S2. Here, the far-field SPL scattering patterns of the ACM with space coding metasurface M1 and M4 are also illustrated in Figure S2. Specifically, it can qualitatively be seen that the maximum SPL of STAM covers from 40 to 47 dB. Compared to that of the ACM with space coding sequences M4, the SPL reduction of STAM in the specular direction is at least 6 dB. The results are confirmed again by the 2D far-field SPL scattering patterns of the ACM and STAM with space coding sequence M4 at the given frequencies in Figure S3.

## Discussion

(1) We have proposed a STAM that can obtain the excellent acoustic backward scattering reduction within the 3D space. The proposed STAM consists of two types of sub-wavelength unit cells with phase difference 180°. Both the theoretical calculations and numerical simulations verified that the proposed STAM can shift the energy of incident acoustic waves to other harmonics and randomly disperse it in the upper half space at the same time via the proposed space-time joint modulation mechanism. Compared with the ACM with spatial coding sequence M1(0000 …), the maximum SPL of the STAM can decrease about 22 dB. The proposed STAMs provide a new method for achieving flexible acoustic wavefront manipulation and further expand the functionality of acoustic coding metasurfaces via fully exploiting the time dimension, which may find potential applications in acoustic communication, holographic imaging, and acoustic camouflage.
(2) It can be pointed that unit cells with time-varying acoustic impedance proposed in this study can be implemented through two distinct methods. The first approach involves designing a membrane structure using piezoelectric materials to achieve the desired time-varying acoustic impedance.^44^ The second approach employs motor-driven dynamic modulation of geometric parameters within the unit cell configuration to attain needed time-varying phases.^50^

### Limitations of the study

This paper primarily proposes and designs a STAM that can obtain the excellent acoustic backward scattering reduction within the 3D space. To enhance the practicality of the STAMs, we will further pursue the following work in the future: the ability of STAM to scatter and curtail acoustic energy is verified by using piezoelectric membrane materials in experiments.

## Resource availability

### Lead contact

Further information and requests for resources should be directed to and will be fulfilled by the lead contact, Wenkang Cao (wkcao@gzu.edu.cn).

### Materials availability

This study did not generate new unique reagents.

### Data and code availability

Data reported in this paper will be shared by the lead contact upon request.
This paper does not report original codes.
Any additional information required to reanalyze the data reported in this paper is available from the lead contact upon request.

## Acknowledgments

This work was supported by the National Natural Science Foundation of China (nos. 52305099, 52305249, and 12104227) and Guizhou Provincial Basic Research Program (Natural Science) (no. ZK (2022) 035).

## Author contributions

Conceptualization, W.C.; S.H. and W.C. jointly edited this manuscript; organize references, K.N. and J.Y.; guidance on research methodology, L.W.; provision of project resources, J.H.

## Declaration of interests

The authors declare no competing interests.

## STAR★Methods

### Key resources table

**Table:** 

REAGENT or RESOURCE	SOURCE	IDENTIFIER
**Software and algorithms**		

MATLAB 2019a	MathWorks	https://www.mathworks.com/matlabcentral/answers/601606-download-matlab-2019a
Origin 2024	OriginLab	https://www.originlab.com/2024
COMSOL 6.2	COMSOL	https://cn.comsol.com/

### Experimental model and study participant details

The COMSOL software was employed to analyze the three-dimensional far-field sound pressure level (SPL) scattering patterns of the Space-time Acoustic Coding Metasurfaces.

### Method details

#### Far-field scattering pattern of the STAM

Under a plane wave with a carrier frequency of $f_{c}$ along -*z* direction, assuming the modulation frequency $f_{0}=1/T_{0}$ is much lower than the carrier frequency $f_{c}$ of incident waves, the far-field scattering pattern of the proposed STAM can be approximately expressed as^52^

(Equation 1) $$f\left(\theta ,\varphi ,t\right)=e^{j2\pi f_{c}t}\sum_{m=1}^{M}\sum_{n=1}^{N}P_{mn}\left(\theta ,\varphi \right)\Gamma_{mn}\left(t\right)e^{jk_{0}}\left[\left(n-1\right)\sin\;\theta \;\sin\;\varphi d_{y}+\left(m-1\right)\sin\;\theta \;\cos\;\varphi d_{x}\right]\text{,}$$

where $P_{mn}\left(\theta ,\varphi \right)$ is the acoustic far-field scattering pattern of the (*m,n*) coding element; *θ* and *φ* are the angle of elevation and azimuth, respectively. $d_{x}$ and $d_{y}$ are periods of coding elements in the *x* and *y* directions, and $k_{0=}2\pi /\lambda_{c}$ ($\lambda_{c}$ is the operating wavelength of the carrier frequency). Besides, $\Gamma_{mn}\left(t\right)$ represents the time modulated reflection coefficient of the (*m,n*) coding element, which is defined as a linear combination of shifted pulse functions within a time interval, which is expressed as^52^

(Equation 2) $$\Gamma_{mn}\left(t\right)=\sum_{v=1}^{L}\Gamma_{mn}^{v}U_{mn}^{v}\left(t\right)\;\left(0<t<T_{0}\right)$$

where $U_{mn}^{v}\left(t\right)$ is a periodic pulse function with a modulation period of $T_{0}$. In one period, it can be expressed as

(Equation 3) $$U_{mn}^{v}\left(t\right)=\left\{ \begin{array}{cc} 1, & \left(v-1\right)\tau \leq t\leq v\tau \\ 0, & otherwise \end{array}\right.$$

where τ *=* $T_{0}/L$ is the pulse width of it and *L* is the length of the time coding sequence, which is defined as a positive integer. $\Gamma_{mn}^{v}$ is the reflection coefficient of the ${\left(m,n\right)}^{th}$ coding element in the time interval (v−1)τ≤t≤vτ. To be specific, $\Gamma_{mn}^{v}$ is given by

(Equation 4) $$\Gamma_{mn}^{v}=A_{mn}^{v}e^{j\varphi_{mn\;}^{v}}$$

where $A_{mn}^{v}$ and $\varphi_{mn}^{v}$ represent the amplitude and phase. By decomposing $U_{mn}^{v}\left(t\right)$ into Fourier series, the Fourier series coefficients $a^{i}$ of the periodic function $\Gamma_{mn}\left(t\right)$ can be shown as^53^

(Equation 5) $$a^{i}=\frac{1}{L}e^{-j\frac{k\pi }{L}}\frac{\sin\left(\frac{k\pi }{L}\right)}{\frac{k\pi }{L}}\sum_{n=0}^{L-1}\Gamma_{n}e^{(\frac{-j2\pi kn}{L})}$$

Here, *i*= …, -2, -1, 0, +1, +2… is the order of harmonics. By adjusting time coding sequence, a serial of reflection coefficients $a^{i}$ can be synthesized to manipulate scattering properties of coding elements.

Finally, combining Equation 1 with Equation 5, the far-field scattering pattern of the STAM at the $i^{th}$ harmonic frequency is

(Equation 6) $$F_{i}\left(\theta ,\varphi \right)=\sum_{m=1}^{M}\sum_{n=1}^{N}P_{mn}\left(\theta ,\varphi \right)e^{\left\{jk_{0}\left[\left(m-1\right)d_{x}\;\sin\;\theta \;\cos\;\varphi +\left(n-1\right)d_{y}\;\sin\;\theta \;\sin\;\varphi \right]\right\}}a_{mn}^{i}$$

where is $a_{mn}^{i}$ Fourier series coefficients of the periodic function $\Gamma_{mn}\left(t\right)$ of the(*m, n*)coding element.

#### Average SPL

In order to further evaluate the intensity distribution of the reflected waves, the average SPL reduction between ACM and STAM in Figure 7D can be calculated by

(Equation 7) $${SPL}_{avg}=\frac{\sum_{i=1}^{n}SPL_{i}}{n}$$

where equal angle interval sampling is utilized. $SPL_{i}$ is the *SPL* in the $i^{th}$ angular direction and *n*=180 is the total number of sampling points.^51^

### Quantification and statistical analysis

The simulation data is produced by COMSOL Multiphysics software. Figures shown in the main text were produced by ORIGIN and Microsoft PowerPoint from the raw data.

### Additional resources

Any additional information about the simulation and data reported in this paper is available from the lead contact on request.
