# Supplementary material for: Space-time acoustic coding metasurfaces with scattering reduction characteristics
Source: iScience. 2025 Apr 9;28(5):112402. doi: 10.1016/j.isci.2025.112402 (PMC12063153; doi:10.1016/j.isci.2025.112402)
Supplement: Document S1. Figures S1–S3 [file mmc1.pdf]

iScience, Volume *28*

Supplemental information

Space-time acoustic coding metasurfaces with scattering reduction characteristics

Sheng He, Wenkang Cao, Kaiping Nie, Jinsong Ye, Jie Hu, and Liting Wu

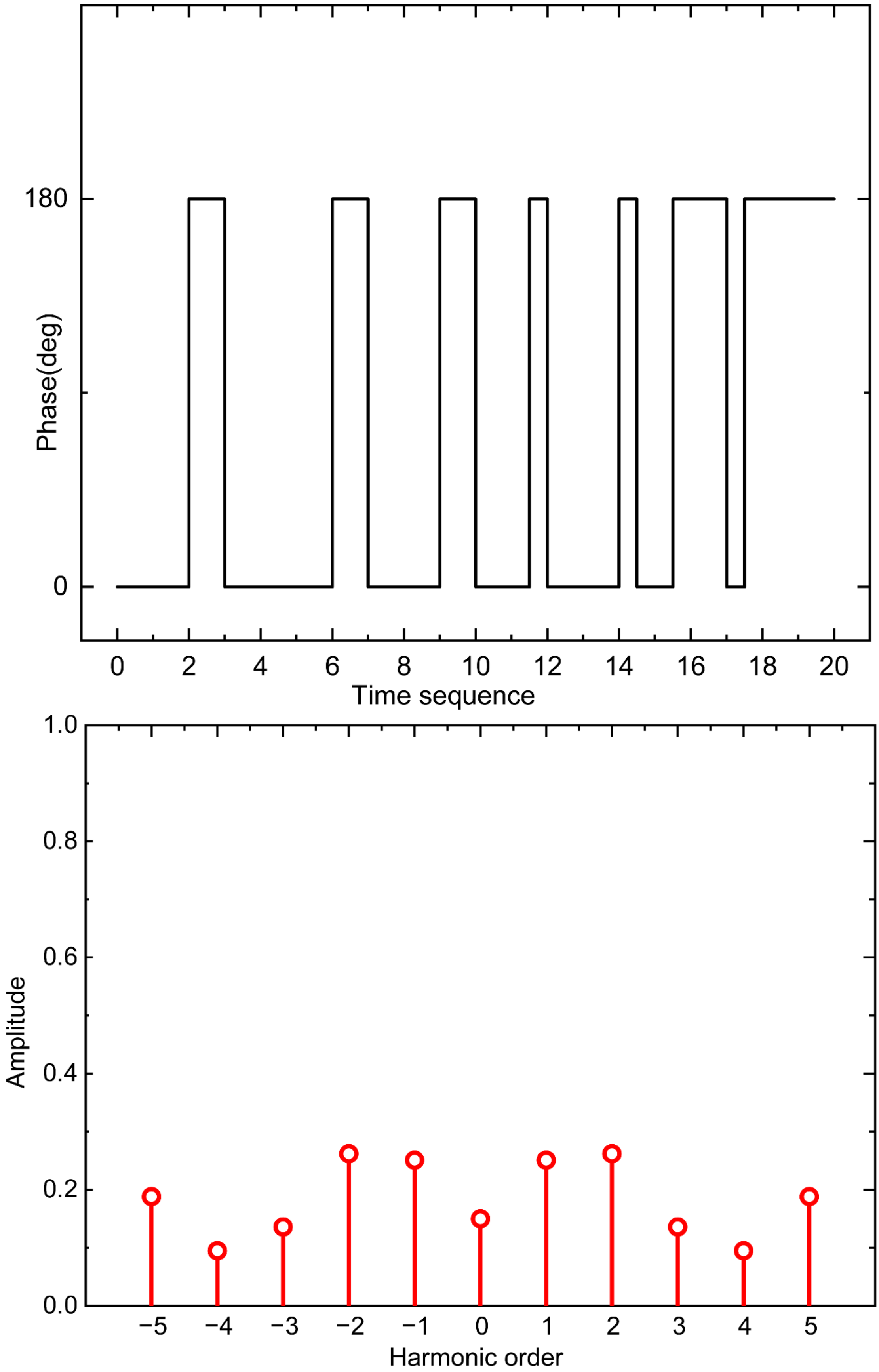

Figure S1. The theoretical analysis of harmonics amplitude distributions of the proposed STAM with longer random time coding sequences.

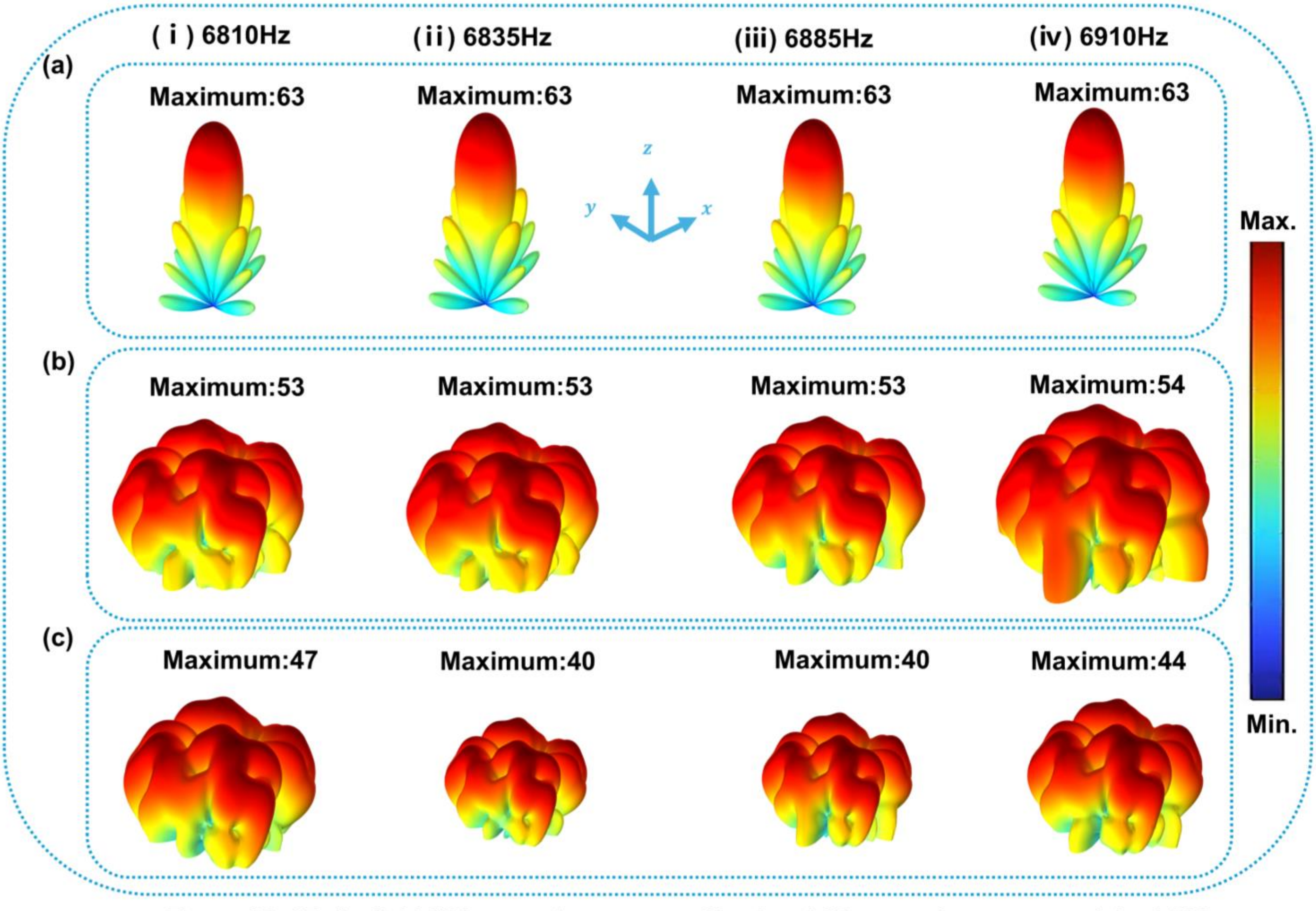

Figure S2. 3D far-field SPL scattering patterns. Simulated 3D scattering patterns of the ACM with (a) space coding sequence M1 and (b) M4. (c) Simulated 3D scattering patterns of the STAM with coding sequence M4 at 6810 Hz, 6835 Hz, 6885 Hz, and 6910 Hz, respectively.

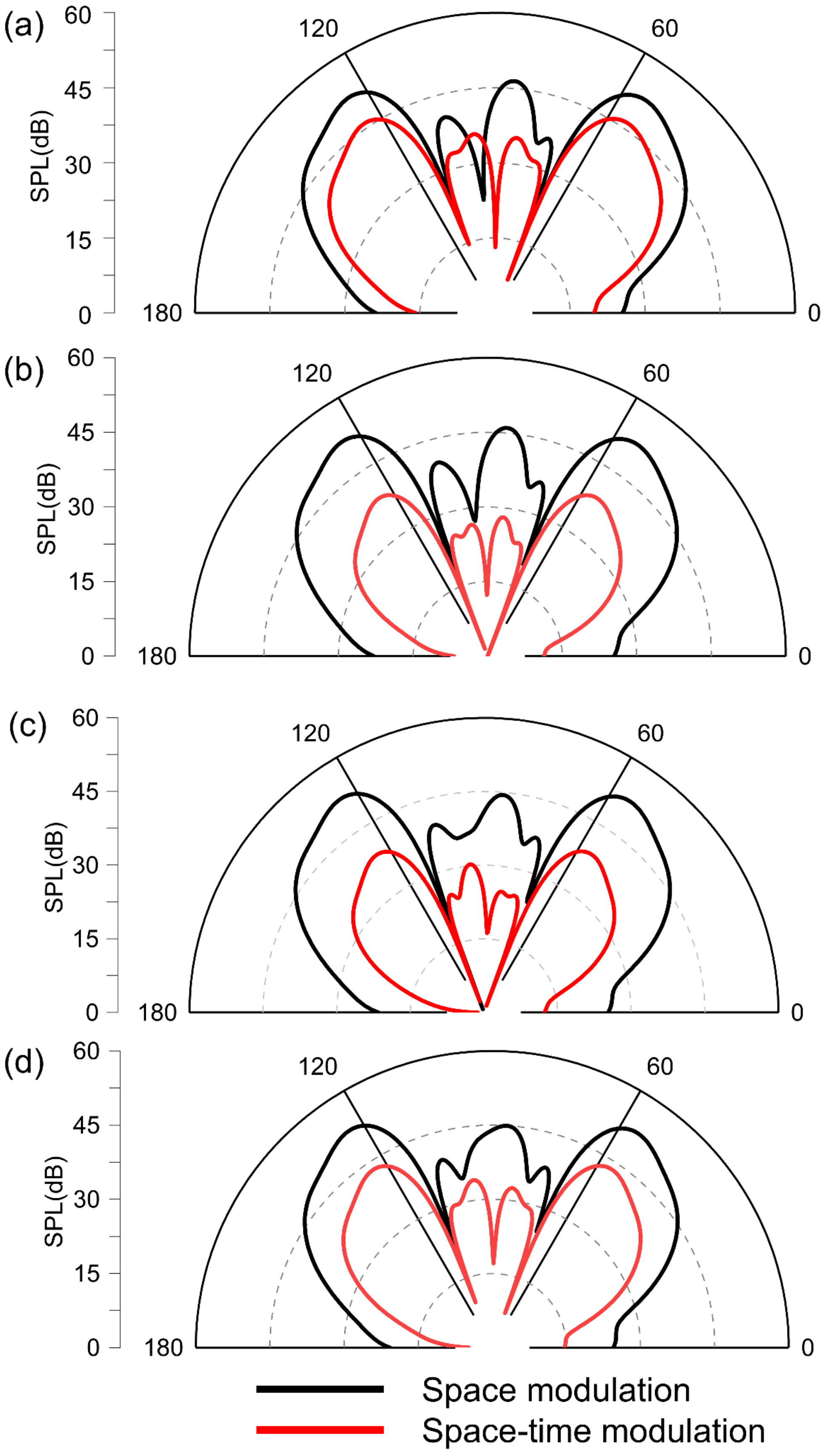

Figure S3. 2D far-field SPL scattering patterns. (a)-(d) The corresponding 2D far-field SPL scattering patterns of the ACM and STAM with space coding sequence M4 in *xoz* direction at 6810 Hz, 6835 Hz, 6885 Hz and 6910 Hz, respectively.
